# Maternal depression does not affect complementary feeding indicators or stunting status of young children (6–23 months) in Northern Ghana

**DOI:** 10.1186/s13104-018-3528-x

**Published:** 2018-06-25

**Authors:** Anthony Wemakor, Habib Iddrisu

**Affiliations:** Department of Nutritional Sciences, School of Allied Health Sciences, University for Development Studies, P O Box TL 1883, Tamale, Ghana

**Keywords:** Stunting, Malnutrition, Complementary feeding indicators, Maternal depression, Northern Ghana

## Abstract

**Objective:**

Maternal depression may affect child feeding practice which is an important determinant of child nutritional status. The objective of this study was to explore the association between maternal depression and WHO complementary feeding indicators [minimum dietary diversity (MDD), minimum meal frequency (MMF) and minimum acceptable diet (MAD)] or stunting status of children (6–23 months) in Tamale Metropolis, Ghana. A community-based cross-sectional study was carried out involving 200 mother–child pairs randomly sampled from three communities in Tamale Metropolis, Ghana.

**Results:**

The prevalence of MDD, MMF, and MAD were 56.5, 65.0, and 44.0% respectively and 41.0% of the children sampled were stunted. A third of the mothers (33.5%) screened positive for depression. Maternal depression did not influence significantly MDD (p = 0.245), MMF (p = 0.442), and MAD (p = 0.885) or children’s risk of stunting (p = 0.872). In conclusion maternal depression and child stunting are prevalent in Northern Ghana but there is a lack of evidence of an association between maternal depression and child feeding practices or nutritional status in this study population. Further research is needed to assess the effect of maternal depression on feeding practices and growth of young children.

**Electronic supplementary material:**

The online version of this article (10.1186/s13104-018-3528-x) contains supplementary material, which is available to authorized users.

## Introduction

About a third of children under five are chronically malnourished in Northern Region, Ghana [1]. Inadequate dietary intake and repeated infections are the two immediate causes of undernutrition but underlying these are a number of determinants including child care [2]. Child care is a complex set of behaviours including child feeding practices [3, 4]. Complementary feeding is the process of introducing foods and liquids to infants when breast milk alone is no longer sufficient to meet their nutritional requirements. The World Health Organisation (WHO) recommends three indicators for assessing the appropriateness of complementary feeding of children (6–23 months) namely minimum meal frequency (MMF), minimum dietary diversity (MDD) and minimum acceptable diet (MAD) [5]. Children receive MMF if they are fed complementary foods the minimum recommended number of times in relation to their age and breastfeeding status. Children receive MDD if they are fed complementary foods from 4 out of 7 food groups. Children who receive both MMF and MDD are said to receive MAD. In 2014, only 21.3% of children under five received MAD in the Northern Region, while 33.3 and 48.4% received MMF and MDD respectively [6].

Maternal depression can negatively affect women’s reproductive roles [7] and although the exact mechanism is not known, it may affect child outcomes postnatally by interfering with mother–child bond and/or provision of child care [8, 9]. Maternal depression has been linked to poor child feeding practices [10] and undernutrition in children in developing countries [11–16].

Children aged 6–23 months are particularly vulnerable to malnutrition because the required amounts of energy and nutrients needed to support their rapid growth may not be provided. Given the high prevalence of child malnutrition in Northern Ghana, research is needed to identify new risk factors in order to inform programme design. Research linking maternal depression and complementary feeding practices or malnutrition in children in Ghana is scanty. We evaluated the association between maternal depression and WHO complementary feeding indicators or nutritional status of children (6–23 months) in Tamale Metropolis, Northern Ghana.

## Main text

### Methods

#### Study design, setting and participants

A population-based cross-sectional study was conducted in a cluster of three communities, Chanshegu, Guunaa yili, and Kabonaa yili, in Tamale Metropolis, Northern Ghana between January and March, 2016. These communities share boundaries and have common socio-cultural characteristics. Prior to the study, a survey was conducted in the study communities to register all children aged 6–23 months and their mothers (n = 239). Using simple random sampling, 200 mother–child pairs were sampled from the 239 registered for the study.

#### Data collection

Data were collected on socio-demographic characteristics and depression status of mothers, and nutritional status of children using questionnaires. The questionnaires were in Dagbani, the local language spoken in the study area. The enumerators were three students of the Department of Nutritional Sciences, UDS.

#### Assessment of complementary feeding indicators

The complementary feeding indicators MDD, MMF and MAD were estimated using data from 24-h recall [17]. In the 24-h dietary recall, the mothers of the children were asked to recall the foods the children ate in the last 24 h prior to the interview. Based on this, the number of food groups children ate from was indicated. Seven standard food groups namely: grains, roots and tubers; legumes and nuts; dairy products; flesh foods; eggs; vitamin A-rich fruits and vegetables; and other fruits and vegetables were used [17]. A child who received foods from ≥ 4 out of the 7 standard food groups during the previous day was classified to have received MDD. A child who was fed at least the recommended number of meals in the last 24 h was classified to have received MMF. A child who received both MDD and MMF was classified to have received MAD.

#### Anthropometry

Anthropometric measurements were performed following standard procedure [18]. Recumbent length was measured using infantometers and the ages of the children were determined from their dates of birth and the dates of the survey. WHO Anthro was used to compute height-for-age z-scores [19]. Height-for-age z-score is derived by comparing the height of the study children to the height of children of the same age and sex (as the study children) in the WHO Child Growth Standards. Children were classified as stunted if they had height-for-age z-scores less than − 2 standard deviations. Stunting is a chronic form of malnutrition manifested as short stature.

#### Assessment of depression

Maternal depression status was determined using Centre for Epidemiologic Studies-Depression (CES-D) scale [20]. CES-D scale comprised of 20 items and asks caregivers to rate how often over the past week they experienced depression-associated symptoms [20]. Each item has four responses. The responses for items 4, 8, 12, and 16 are scored 3, 2, 1 and 0 respectively whiles the responses for all other items are reverse scored. The total CES-D score is obtained by summing up the scores for the 20 items. A cut-off of ≥ 20 [21] was used to classify the mothers as depressed.

#### Statistical analysis

Data were analyzed using Stata (version 13). Descriptive statistics were computed, frequencies and percentages for categorical variables, and means and standard deviations for continuous variables. Pearson Chi square (or Fisher’s exact) test was used to compare the socio-demographic characteristics of the mothers with and without depression. Logistic regression analysis was used to estimate the odds of receiving MMF, MDD, and MAD by children of depressed mothers compared to children of non-depressed mothers. Similarly, the risk of stunting in children of depressed mothers compared to children of non-depressed mothers was estimated. In all statistical tests, a p value < 0.05 was considered statistically significant.

#### Ethics

Ethical clearance (Protocol Number 26-2015) was obtained for the study from the Joint Ethical Review Committee of School of Medicine and Health Sciences and School of Allied Health Sciences, University for Development Studies, Tamale, Ghana. Also, informed consent was obtained from the subjects before they were enrolled into the study.

### Results

The mothers’ age ranged from 18 to 41 years with a mean of 27.0 (± 5.1) years and most (45.5%) were in the 26–30 years age group (Table 1). The majority of the mothers had no education (58.0%), and were traders (69.0%); a greater majority belonged to Dagomba ethnic group (88.5%), practised Islamic religion (97.0%) and were married (94.5%).

**Table 1:** Socio-demographic characteristics of mothers and children

Characteristic	Frequency (n = 200)	Percent
Mothers		
Age group (years)		
< 20	8	4.0
20–24	50	25.0
25–29	89	44.5
30–34	38	19.0
35+	15	7.5
Highest educational level completed		
No education	116	58.0
Primary	19	9.5
Junior High School/Middle school	39	19.5
Senior High School/Vocational school	17	8.5
University	9	4.5
Marital status		
Married	189	94.5
Single, never married	10	5.0
Divorced/separated/widowed	1	0.5
Religion		
Islamic	194	97.0
Christian	6	3.0
Occupation		
Trader	138	69.0
Farmer	18	9.0
Teacher	6	3.0
Others	38	19.0
Ethnicity		
Dagomba	177	88.5
Gonja	10	5.0
Mamprusi	9	4.5
Others	4	2.0
Children		
Age group (month)		
6–11	82	41.0
12–23	118	59.0
Sex		
Male	86	43.0
Female	114	57.0

The ages of the children ranged from 6 to 23 months with a mean of 13.4 (± 5.3) months, and most were in the 12–23 months age group (59.0%), and were females (57.0%) (Table 1). The mean weight and height of the children were 8.9 (± 2.4) kg, and 71.5 (± 6.5) cm respectively. About 40% of the children had stunting (41.0%) and stunting prevalence rate was higher for older children (12–23 months) than younger ones (6–11 months) and for males than females (Table 2). With respect to the indicators of complementary feeding, 56.5, 65.0 and 44.0% of the children received MDD, MMF and MAD.

**Table 2:** Mean height-for-age z-score and prevalence of stunting in children 6–23 months, by age and sex

Height-for-age z-score	Number	Mean	SD	Prevalence of stunting (%)
Age group (months)				
6–11	82	− 1.46	1.53	36.9
12–23	118	− 1.82	1.35	44.1
Sex				
Male	86	− 2.08	1.35	51.2
Female	114	− 1.36	1.42	33.3
All	200	− 1.67	1.43	41.0

About a third of the mothers (33.5%) screened positive for depression. There were no significant differences in the distribution of the socio-demographic characteristics of the mothers with and without depression (data not shown) (Additional file 1).

The likelihood of obtaining MMF, MDD and MAD by children of depressed mothers was determined by comparing them to children of non-depressed mothers. Children of mothers with and without depression had similar likelihoods of receiving the three indicators of complementary feeding—MMF [crude odds ratio (COR): 1.28, 95% confidence interval (CI) 0.68–2.39], MDD (COR: 0.70, 95% CI 0.39–1.27) and MAD (COR: 0.96, 95% CI 0.53–1.73) (Table 3). Similarly, children belonging to mothers with and without depression had comparable risks of stunting (Table 3).

**Table 3:** Comparing complementary feeding indicators and nutritional status of children of mothers with and without depression

Characteristic	Children of non-depressed mothers (%), n = 133	Children of depressed mothers (%), n = 67	Depression status of mother	Odds ratio (95% CI)	p value
Minimum meal frequency					0.442
No	36.8	31.3	Non-depressed	1.00	
Yes	63.2	68.7	Depressed	1.28 (0.68–2.39)	
Minimum dietary diversity					0.245
No	40.6	49.3	Non-depressed	1.00	
Yes	59.4	50.7	Depressed	0.70 (0.39–1.27)	
Minimum acceptable diet					0.885
No	55.6	56.7	Non-depressed	1.00	
Yes	44.4	43.3	Depressed	0.96 (0.53–1.73)	
Stunting					0.872
No	59.4	58.2	Non-depressed	1.00	
Yes	40.6	49.8	Depressed	1.05 (0.58–1.91)	

### Discussion

We explored the role of child feeding practices in mediating the effects of maternal depression on the growth of young children. We found similar rates of WHO complementary feeding indicators and undernutrition among children of mothers with and without depression, suggesting maternal depression does not affect child feeding practices and nutritional status in this study population. To our knowledge this is the first study to link maternal depression to WHO complementary feeding indicators.

The percentage of stunted children was higher than the average for Northern Region (33.1%) [1]. This may reflect the exposure of these children to sub-optimal breastfeeding and complementary feeding practices and repeated infections [22, 23]. Stunting which reflects accumulation of exposure to sub-optimal feeding practices and repeated infections was more frequent in older children than younger ones and in males than females as reported in other studies also [24–26]. The possible reasons for this finding include increased morbidity rate in early infancy [27–29], and greater biologically programmed growth trajectory for male infants which causes higher demands for most nutrients that may not be met [30].

A high percentage of the children (65.0%) received MMF, 56.0% received MDD and less than half (44%) received MAD. These figures are higher than the regional averages [6] probably because most mothers were engaged in foodstuff petty trade. In most cases, children are fed the required number of times with less dietary diversity or vice versa and the result in each case is a diet inadequate to meet the nutritional requirements of children and predisposes them to malnutrition [2].

Interestingly, we obtained higher prevalence rates for both complementary feeding indicators and stunting compared to figures for Northern Region. Given the higher prevalences of MMF, MDD, and MAD observed in our study, one would have expected a lower rate of stunting but this was not the case reflecting a lack of association between complementary feeding indicators and child nutritional status, a finding also reported by another study [31]. An alternative explanation is the unavailability of other important determinants of child nutritional status such as preventive health services. This reinforces the fact that child nutritional status is a product of food and non-food factors, both of which must be available for proper growth and development of children.

A higher prevalence of maternal depression was estimated (33.5%) than previously reported for a population of mothers in Ghana (3.8–27.8%) [11, 13, 32–36]. Among these studies the highest rate (27.8%) was reported for mothers in Northern Ghana [11] while lower rates were reported for southern Ghana (3.8–11.3%). This reflects the high socio-economic disparity between southern and northern Ghana [37] and the correlation between depression and low socio-economic status [38, 39].

Maternal depression can affect child feeding practices and nutritional status of young children.

Our study does not agree with two studies that reported negative effect of depression on breastfeeding [12, 40]. Some studies link maternal depression and undernutrition in children [11, 14–16] but a similar study like ours does not [41]. The possible reasons for the lack of associations between maternal depression and complementary feeding indicators or child nutritional status in our study population include the nonexistence of these associations in the study population, a lack of sufficient power by our study to detect these associations if they exist or the sharing of child care resources by families. Child care support is known to lessen the impact of maternal mental illness on child growth indicators [42]. In most Ghanaian families in rural areas, all adults in the household contribute to child care and this may have happened in the study area to ensure a mother’s depression status does not influence the feeding or nutritional status of her children.

## Conclusions

Both maternal depression and child undernutrition are prevalent in Northern Ghana. We did not find evidence of an association of maternal depression with child feeding practices or nutritional status. Further research is needed to verify the influence of maternal depression on child feeding practices and nutritional status.

## Limitations

- The translated CES-D scale used in data collection was not validated for Ghanaian caregivers and we did not include a clinical diagnostic interview to serve as a “gold standard” against which it could be compared.
- The use of one 24-h dietary recall data for estimating the indicators of complementary feeding may not provide information on the usual dietary pattern.
- We used a cross-sectional study design which is not appropriate for studying cause-and-effect relationships.

## Additional files

**Additional file 1.** Comparison of socio-demographic characteristics of mothers with and without depression. This is a table comparing socio-demographic information of mothers with and without depression.

**Additional file 2.** Study dataset. This is the dataset analysed for the manuscript.

## Abbreviations

CES-D: Centre for Epidemiologic Studies Depression
CI: confidence interval
COR: crude odds ratio
MAD: minimum acceptable diet
MDD: minimum dietary diversity
MMF: minimum meal frequency
SD: standard deviation
UDS: University for Development Studies
WHO: World Health Organisation
