# Supplementary material for: Maternal depression does not affect complementary feeding indicators or stunting status of young children (6–23 months) in Northern Ghana
Source: BMC Res Notes. 2018 Jun 25;11:408. doi: 10.1186/s13104-018-3528-x (PMC6019211; doi:10.1186/s13104-018-3528-x)
Supplement: Supplementary file 1 — Additional file 1. Comparison of socio-demographic characteristics of mothers with and without depression. This is a table comparing socio-demographic information of mothers with and without depression. [file 13104_2018_3528_MOESM1_ESM.docx]

**Additional file 1: Comparison of socio-demographic characteristics of mothers with and without depression**

| **Characteristic** | **Number** | **No depression (n=133)** | | **Depression (n=67)** | | **P-value** |
| --- | --- | --- | --- | --- | --- | --- |
|  |  | **Frequency** | **Percent** | **Frequency** | **Percent** |  |
| **Age group (years)** |  |  |  |  |  | 0.697 |
| ≤24 | 58 | 36 | 62.1 | 22 | 37.9 |  |
| 25-29 | 89 | 63 | 70.8 | 26 | 29.2 |  |
| 30-34 | 38 | 24 | 63.2 | 14 | 36.8 |  |
| ≥35 | 15 | 10 | 66.7 | 5 | 33.3 |  |
| **Education** |  |  |  |  |  | 0.209 |
| No education | 116 | 73 | 62.9 | 43 | 37.1 |  |
| Some education | 84 | 60 | 71.4 | 24 | 28.6 |  |
| **Marital status** |  |  |  |  |  | 0.045^1^ |
| Currently married | 189 | 129 | 68.3 | 60 | 31.7 |  |
| Not currently married^2^ | 11 | 4 | 36.4 | 7 | 63.6 |  |
| **Religion** |  |  |  |  |  | 0.182^1^ |
| Islamic | 194 | 127 | 65.5 | 67 | 34.5 |  |
| Christian | 6 | 6 | 100.0 | 0 | 0.0 |  |
| **Occupation** |  |  |  |  |  | 0.470 |
| Trader | 138 | 94 | 68.1 | 44 | 31.9 |  |
| Others | 62 | 39 | 62.9 | 23 | 37.1 |  |
| **Ethnicity** |  |  |  |  |  | 0.890 |
| Dagomba | 177 | 118 | 66.7 | 59 | 33.3 |  |
| Others | 23 | 15 | 65.2 | 8 | 34.8 |  |
| ^1^Fisher’s exact test was conducted.  ^2^Not currently married includes mothers who were never married, and mothers who were divorced or widowed. | | | | | | |
